# The effect of distal coronary embolization on infarct size in a porcine acute myocardial infarction model

**DOI:** 10.1186/1532-429X-15-S1-P198

**Published:** 2013-01-30

**Authors:** Reuben M Thomas, Nilesh R Ghugre, Beiping Qiang, Michelle Ladouceur-Wodzak, Xiuling Qi, Rafael Wolff, Azriel Osherov, Mansoor Husain, Graham Wright, Bradley H Strauss

**Affiliations:** 1 Platform of Biological Sciences, Sunnybrook Research Institute, Toronto, ON, Canada; 2 Department of Laboratory Medicine and Pathobiology, University Of Toronto, Toronto, ON, Canada; 3 Platform of Physical Sciences, Sunnybrook Research Institute, Toronto, ON, Canada; 4 Schulich Heart Research Program, Sunnybrook Health Sciences Centre, Toronto, ON, Canada; 5 Toronto General Hospital Research Institute, University Health Network, Toronto, ON, Canada; 6 Department of Medical Biophysics, University Of Toronto, Toronto, ON, Canada

## Background

Reperfusion no-reflow (R-NR), characterized by TIMI 0/1 flow grade, is a phenomenon that occurs in up to 20% of percutaneous coronary intervention cases for acute myocardial infarction (AMI). R-NR involves reduced myocardial tissue perfusion in the presence of a reperfused and patent epicardial coronary artery. This phenomenon is associated with worse patient outcome and increased mortality. Potential contributing factors of R-NR include the initial ischemic insult, injury as a result of reperfusion, and possibly distal coronary embolization (DCE) of thrombotic material. The objective of this study was to develop a novel large animal model to investigate the effects of DCE in the setting of ischemia-reperfusion using microthrombi derived from autologous porcine blood.

## Methods

3.0 T CMR was performed on 25-30kg female Yorkshire pigs to determine baseline values of cardiac function, as well as the myocardial perfusion territory at risk using direct intracoronary injection of dilute Gadolinium-DTPA. Pigs were then subjected to balloon occlusion mediated myocardial ischemia distal to the second diagonal branch of the left anterior descending coronary artery (LAD) for 60 minutes. Following reperfusion, intracoronary injection of sterile saline (n=4) or microthrombi particles to simulate DCE (n=7) was performed. Epicardial coronary blood flow was assessed at each stage using x-ray angiography. At 72 hours following ischemia-reperfusion, CMR was used to assess changes in cardiac function, extent of edema/hemorrhage using T2/T2* relaxation times, evaluate infarct size, and to determine the presence/absence of microvascular obstruction (MVO). Myocardial tissue samples were subsequently collected for biochemical and histological analysis.

## Results

Administration of 5-10 mg of microthrombi following 60 minute ischemia was sufficient to create no-reflow conditions following ischemia in all seven pigs. Early gadolinium enhancement imaging at 72 hours post-ischemia revealed the presence of MVO in 6 of 7 pigs which received microthrombi. MVO was not visible in 3 out of 4 control pigs administered saline following ischemia-reperfusion. Volumetric late-gadolinium enhancement images were used to evaluate infarct size. 11.7% of total LV volume (38.8% of perfusion territory) was infarcted for control, saline administered pigs. Pigs that received microthrombi had an infarct size of 12.9% of total LV volume (43.2% of perfusion territory). Global cardiac functional parameters did not reveal significant changes compared to baseline. Regional alterations in wall thickness/wall motion in addition to T2/T2* relaxation times were observed. Quantitative analyses of these parameters are in progress.

## Conclusions

Our porcine model is the first to combine myocardial ischemia with distal coronary embolization to more closely replicate the human condition. Our preliminary results suggest that DCE is capable of inducing no-reflow which results in microvascular obstruction as determined by MRI.

## Funding

Heart and Stroke Foundation of Ontario,

Canadian Institutes of Health Research

